# Genetic Evidence for the Benefits and Risks of Glucose-Lowering Drugs on Cardiovascular-Kidney-Metabolic Syndrome: A Drug-Target Mendelian Randomization Study

**DOI:** 10.7150/ijms.133077

**Published:** 2026-05-11

**Authors:** Jian Lu, Shuaigang Sun, Xinru Shang, Shimin Jiang, Zekai Deng, Shunwei Wang, Chenping Wei, Jiaqi Hu, Wenge Li

**Affiliations:** 1 Department of Nephrology, China-Japan Friendship Hospital, Beijing, China.; 2 Department of Nephrology, Capital Medical University China-Japan Friendship School of Clinical Medicine, Beijing, China.; 3 Department of Nephrology, Peking University China-Japan Friendship School of Clinical Medicine, Beijing, China.; 4 School of Basic Medical Sciences, Capital Medical University, Beijing, China.; 5 School of Public Health, Capital Medical University, Beijing, China.; 6 School of Healthcare Management, Tsinghua Medicine, Tsinghua University, Beijing, China.; 7 Institute for Hospital Management, Tsinghua University, Beijing, China.

**Keywords:** cardiovascular-kidney-metabolic syndrome, Mendelian randomization, glucose-lowering drugs, SGLT2 inhibitors, GLP-1 receptor agonists

## Abstract

**Background:**

To explore the potential benefits and risks of glucose-lowering drugs on cardiovascular-kidney-metabolic (CKM) syndrome outcomes using drug-target Mendelian randomization (MR).

**Methods:**

Genetic instruments for eight glucose-lowering drugs were identified from variants associated with glycated hemoglobin (HbA1c) and drug target gene expression. We employed two-sample MR and meta-analysis to estimate associations with nine CKM syndrome-related diseases, including coronary artery disease (CAD), myocardial infarction (MI), stroke, heart failure (HF), atrial fibrillation (AF), venous thromboembolism (VTE), peripheral artery disease (PAD), chronic kidney disease (CKD), and metabolic syndrome (MetS), using data from UK Biobank, FinnGen, and other large GWAS consortia. Supplementary analyses included summary-data-based MR (SMR) and colocalization.

**Results:**

Genetic proxies for sodium—glucose cotransporter 2 inhibitors (SGLT2i) were associated with reduced risks of CAD (OR 0.85, 95% CI 0.73—0.98), HF (OR 0.66, 95% CI 0.44—0.98), and MetS (OR 0.64, 95% CI 0.50—0.82). GLP-1 receptor agonists (GLP-1RA) were linked to lower risks of CAD (OR 0.90, 95% CI 0.84—0.96), HF (OR 0.92, 95% CI 0.86—0.99), and CKD (OR 0.87, 95% CI 0.79—0.95). Metformin showed protective association with CAD (OR 0.51, 95% CI 0.40—0.66) and PAD (OR 0.99, 95% CI 0.99—1.00). Sulfonylureas showed a modest association with reduced CAD (OR 0.98, 95% CI 0.96—1.00). Conversely, insulin was associated with a higher risk of MetS (OR 1.07, 95% CI 1.02—1.13), and thiazolidinediones (TZDs) with an increased risk of HF (OR 1.12, 95% CI 1.05—1.20). SMR provided additional evidence for protective roles of *GLP1R* and *VEGFA* for CAD, *KCNJ11* for HF*, ABCC8* for HF and AF, and *PRKAB1* for multiple cardiovascular outcomes. The detrimental effects of *INSR* on MetS and *SERPINE1* on CKD and MetS were also verified.

**Conclusions:**

This study provides genetic evidence revealing the potential benefits and risks of certain glucose-lowering drugs in the management of CKM syndrome. These findings may inform target validation, drug repurposing, and personalized therapies for CKM syndrome.

## Introduction

Growing recognition has been given to the complex pathological links among metabolic dysfunction, cardiovascular disease (CVD), and chronic kidney disease (CKD). These interconnected mechanisms—insulin resistance, advanced glycation, dyslipidemia, chronic inflammation, and endothelial dysfunction—contribute to multi-organ impairment and increase the risk of adverse cardiovascular and renal outcomes 1. In 2023, the American Heart Association (AHA) formally defined this cluster as cardiovascular-kidney-metabolic (CKM) syndrome and further staged its progression from 0 to 4 2. Epidemiological evidence indicates that nearly 90% of U.S. adults meet criteria for stage 1 or higher, with approximately 15% in advanced stages 3. Similar burdens are observed among middle-aged and older Chinese populations 4. Patients with CKM syndrome face markedly increased premature mortality 5, and CKM status is now recognized as a key determinant of all-cause mortality 6. Together, these findings highlight CKM syndrome as a pressing global health challenge and underscore the urgent need for therapeutic strategies that provide broad protection across metabolic, cardiovascular, and renal domains.

Diabetes is a major driver of CKM syndrome 7. In 2021, the International Diabetes Federation estimated that approximately 537 million adults worldwide have diabetes, accounting for 11% of the global population 8. A cohort study of 1.2 million type 2 diabetes (T2D) patients across six countries found that, among those without pre-existing CVD or CKD, 24% of initial events were heart failure (HF), stroke (16%), myocardial infarction (MI) (14%), peripheral artery disease (PAD) (10%), and CKD (36%) 9. Additionally, a study of 530,747 U.S. adults with T2D found that isolated T2D without other CKM conditions was rare (6.4%), with around 51% having three or more additional CKM-related conditions 10. These findings suggest that glucose-lowering drugs may have consequences far beyond glycemic control, shaping outcomes across the CKM spectrum.

In recent years, antidiabetic agents have been recognized not only for glucose-lowering efficacy but also for cardiovascular and renal benefits. Landmark randomized controlled trials (RCTs) 11-21 have demonstrated that sodium-glucose cotransporter-2 inhibitors (SGLT2i) and glucagon-like peptide-1 receptor agonists (GLP-1RA) substantially reduce risks of major adverse cardiovascular events, heart failure hospitalization, and kidney disease progression—providing “triple protection” across metabolic, cardiovascular, and renal axes. These findings have shifted clinical practice guidelines and positioned SGLT2i and GLP-1RA as cornerstone therapies in T2D and atherosclerotic cardiovascular disease (ASCVD) 22-23. Nevertheless, the underlying mechanisms remain incompletely understood, and evidence for other glucose-lowering drug classes such as insulin and its analogues, metformin, sulfonylureas, α-glucosidase inhibitors (AGIs), thiazolidinediones (TZDs), dipeptidyl peptidase-4 inhibitors (DPP4i) remains inconsistent and even harmful in certain contexts 24-27. Moreover, traditional clinical trials are limited by selective enrollment and potential confounding, leaving knowledge gaps about long-term efficacy and safety.

Mendelian randomization (MR) offers a powerful complementary approach by leveraging genetic variants as instrumental variables to infer causal effects while minimizing bias from confounding and reverse causation 28. Extending this framework, drug-target MR utilizes genetic proxies of therapeutic targets to evaluate the likely effects of pharmacological modulation 29. In this study, we applied a two-sample drug-target MR design across large-scale GWAS datasets of European ancestry to systematically evaluate the associations of eight major glucose-lowering drug classes on nine CKM syndrome-related outcomes. We further integrated summary-data-based MR (SMR) and colocalization analyses to validate gene-trait expression and distinguish pleiotropy from shared genetic causation. This integrative framework aims to refine the understanding of drug mechanisms and provide genetic evidence for potential therapeutic and repurposing strategies in the context of CKM syndrome.

## Methods

### Identification and validation of glucose-lowering drug targets

**Figure 1:**
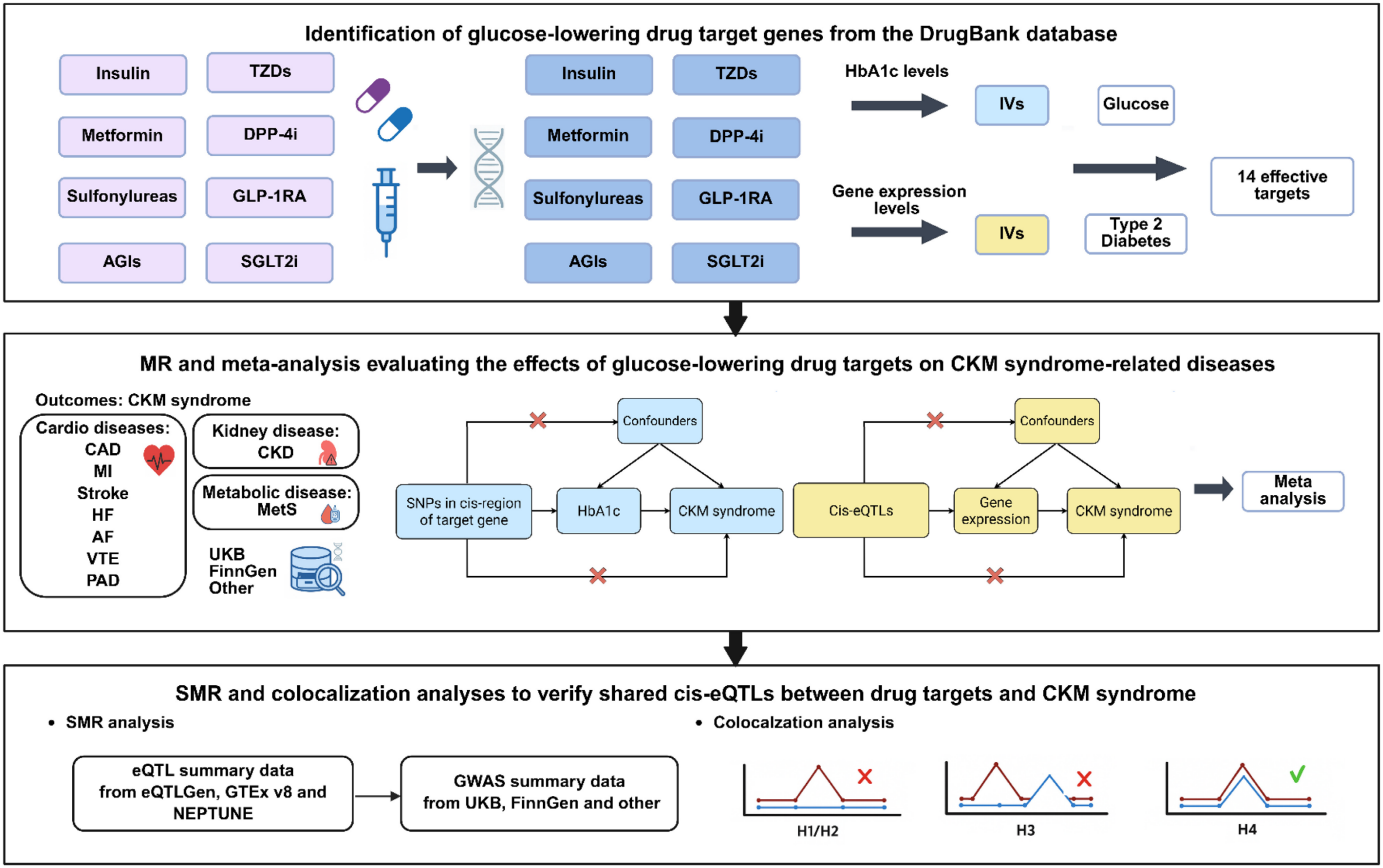
Overall workflow of this study.

The overall workflow of this study is illustrated in **Figure 1**. Briefly, we conducted the analysis in two stages: (i) a two-sample MR analysis to assess the causal effects of genetic proxies for glucose-lowering drug targets on a spectrum of CKM syndrome outcomes, and (ii) SMR and colocalization analyses to evaluate whether any detected drug-disease associations were mediated through gene expression, thereby serving as complementary validation.

We comprehensively identified genetic targets of eight commonly prescribed glucose-lowering drug classes using the DrugBank pharmacogenomics database (https://go.drugbank.com/) **(Table S1)**: insulin and its analogues, metformin, sulfonylureas, AGIs, TZDs, DPP4i, GLP-1 RAs, and SGLT2i. The pharmacological mechanisms and chromosomal locations of these targets are presented in **Table S2**.

Instrumental variables (IVs) were extracted from the GWAS of glycated hemoglobin (HbA1c) in the UK Biobank population 30, using the thresholds of *P* < 5 × 10⁻⁸ and r² < 0.2 31. For two adjacent drug targets located within overlapping cis regions and exerting effects in the same mechanistic direction, we combined them and denoted them with a slash (e.g., *ABCC8/KCNJ11* and *ABCC9/KCNJ8*). Furthermore, eQTLs for drug-target genes were retrieved from the eQTLGen Consortium (https://www.eqtlgen.org), with only cis-eQTLs located within 500 kb of the target gene included as IVs 32.

To ensure consistency with pharmacological mechanisms, we adjusted the directionality of effect estimates for all genetic instruments at the individual target gene level prior to downstream analyses. Specifically, for genetic proxies corresponding to agonists or mechanisms of uncertain directionality (e.g., modulators, agonists, activators, substrates, or unknown/other mechanisms), we retained the original β coefficients. Conversely, for inhibitors (e.g., blockers, inhibitors, antagonists, or inverse agonists), we applied the negative β values. Then, all validated and direction-aligned SNPs corresponding to the various targets of a specific drug class (e.g., pooling *SLC5A1* and *SLC5A2* for SGLT2i) were combined into a single composite IV set in order to reflect the composite action of drug classes rather than isolated targets. To minimize weak IV bias, we calculated F-statistics using the formula F = R² × (N - 2) / (1 - R²), where R² = β² / (β² + SE² × (N - 1)), excluding IVs with F-statistics < 10** (Table S3)** 33. Finally, to validate the robustness of the selected IVs, we employed four independent GWAS datasets related to blood glucose levels and T2D as positive controls 31,33. Given the established glucose-lowering effects of these drugs, only drug-gene target pairs showing significant associations consistent with reduced glycemia (β < 0, *P* < 0.05) were advanced to subsequent analyses.

### Outcome data of the CKM syndrome

We focused on nine major clinical outcomes that represent the core components of the CKM syndrome, including coronary artery disease (CAD), MI, stroke, HF, atrial fibrillation (AF), venous thromboembolism (VTE), PAD, CKD, and metabolic syndrome (MetS). For each outcome, we obtained summary-level GWAS statistics from three independent data sources to enhance robustness and reduce dataset-specific bias. The primary GWAS datasets were obtained from the UK Biobank, FinnGen, and other large disease-specific international consortia, such as CKDGen for CKD and GIGASTROKE for stroke, or GWAS meta-analyses. However, due to the unavailability of FinnGen GWAS data for MetS, only two GWAS datasets were used. All data were derived from previously published GWAS studies, and detailed information is provided in **Table S4**.

### Evaluation of sample overlap bias

Given that both the HbA1c exposure and several outcome studies included participants from the UK Biobank, potential bias and Type 1 error rate inflation associated with varying levels of sample overlap rates were estimated using the mrSampleOverlap R package (https://www.levin-lab.org/mrSampleOverlap/), which strictly implements the analytic formulae derived by Burgess et al. for two-sample MR 34.

### Two-sample MR analysis and meta-analysis

Two-sample MR was applied to evaluate the causal effect of genetically proxied glucose-lowering drug targets on outcomes, rather than the effects of blood glucose changes. As illustrated in **Figure 1**, MR design relies on three core assumptions: (i) IVs are strongly associated with the exposure; (ii) IVs are independent of potential confounders; and (iii) IVs affect the outcome only through the exposure of interest.

The inverse variance-weighted (IVW) method was used as the primary MR approach, under either a random-effects or fixed-effects model depending on the results of heterogeneity testing 35. To assess the robustness of the findings, we additionally performed sensitivity analyses using MR-Egger regression, weighted median, simple mode, and weighted mode methods. For drug targets with fewer than three valid SNP instruments, causal estimates were obtained using the Wald ratio method. Heterogeneity was evaluated using Cochran's Q statistic derived from both IVW and MR-Egger models. Potential horizontal pleiotropy was examined by testing the intercept term from MR-Egger regression. All MR analyses were performed using the TwoSampleMR package in R (https://mrcieu.github.io/TwoSampleMR/) with default settings.

To obtain an overall causal effect estimate, we performed a meta-analysis of the causal estimates derived from the primary MR method (IVW for ≥ 3 SNPs or the Wald ratio for < 3 SNPs) for each drug target-outcome pair. Heterogeneity was evaluated using Cochran's Q statistic and the I² index. A fixed-effects model was applied when no significant heterogeneity was observed (*P* ≥ 0.05 and I² ≤ 50%), whereas a random-effects model was used when significant heterogeneity was present (*P* < 0.05 and I² > 50%). The pooled estimates from the meta-analysis were utilized to quantify the magnitude of the final causal effects. However, if only one reliable MR result was available, the causal inference was based on that single estimate. Given the broadly exploratory nature of this study, the false discovery rate (FDR) was applied for multiple testing correction. Specifically, an FDR < 0.05 indicated a significant causal relationship between the exposure and the outcome, while a nominal *P* < 0.05 with an FDR ≥ 0.05 was considered suggestive.

### Summary-data-based MR

To further examine the associations between gene expression of eight glucose-lowering drug targets and the risk of CKM syndrome, we applied the SMR method, using the most significantly associated cis-eQTL SNP as the primary IV, and effect estimates were expressed as OR for disease risk per-SD increase in genetically predicted expression of each drug target gene. To complement the whole-blood cis-eQTL from eQTLGen, we additionally integrated tissue-specific cis-eQTLs from cardiac tissues (left ventricle and atrial appendage) from genotype-tissue expression (GTEx) version 8 36, and renal tissues (glomeruli and tubulointerstitium) from NEPTUNE 37. The HEIDI (heterogeneity in dependent instruments) test was conducted to distinguish true causal associations from those potentially driven by linkage disequilibrium (LD) between the eQTL SNP and another causal variant. A HEIDI test *P*-value < 0.05 was considered indicative that the observed association might be attributable to LD rather than gene expression itself. SMR analyses were performed using the default parameters (https://yanglab.westlake.edu.cn/software/smr/#SMR&HEIDIanalysis).

### Colocalization analysis

To explore whether the associations between specific gene expression and CKM syndrome outcomes were attributable to a shared causal genetic variant, we performed a Bayesian colocalization analysis using the coloc R package (http://cran.r-project.org/web/packages/coloc) with default prior settings (P1 = 1 × 10⁻⁴, P2 = 1 × 10⁻⁴, P12 = 1 × 10⁻⁵) 38. Evidence for colocalization was considered strong to indicate a high probability of a shared causal variant when the posterior probability for hypothesis 4 (PPH4) exceeded 0.8.

## Results

### Identification and validation of glucose-lowering drug targets

Based on HbA1c levels, we identified 18 potential target genes across eight classes of glucose-lowering drugs: Insulin (*LRP2*), Metformin (*GPD1*), Sulfonylureas (*ABCC8/KCNJ11*, *ABCC9/KCNJ8*, *ABCB11*, *CPT1A*, *VEGFA*, *INS*, *KCNJ1*), AGIs (*GANC*), TZDs (*PPARG*, *ESRRA*, *SERPINE1*, *SLC29A1*, *RXRB*), GLP-1RA (*GLP1R*), and SGLT2i (*SLC5A2*, *SLC5A1*). Bias due to sample overlap from the UK Biobank participants included in both the HbA1c exposure and several outcome GWASs was mathematically negligible across a wide range of assumed observational effect sizes: for instance, in the analysis of *LRP2* on AF, at 80.5% sample overlap, bias was estimated to be 0.001 for an observational OR of 1.3, and 0.002 for an OR of 1.6 (**Table S5**). Among these, nine targets were significantly associated with reduced glycemia or reduced risk of T2D and were retained for further analyses **(Table S6)**. It should be noted that no valid IVs meeting our stringent selection criteria were available for DPP4i, therefore, this drug class was not included in the analyses.

Based on gene expression levels, we initially identified 26 potential target genes from the eQTLGen Consortium: Insulin (*INSR, IGF1R, IGFBP7*), Metformin (*ETFDH, PRKAB1, ACACB*), Sulfonylureas (*KCNJ11, ABCA1, CPT1A, TRPM4, VEGFA*), AGIs (*MGAM, GAA, GANAB, GANC*), TZDs (*PPARG, ESRRA, PPARD, PPARA, GSTP1, SERPINE1, SLC29A1, RXRA*), DPP4i (*DPP4*), GLP-1RA (*GLP1R*), and SGLT2i (*SLC5A2*). However, only five target genes passed positive control analyses.

Subsequently, after adjusting effect directions according to drug-target mechanisms, we combined all validated IVs within each drug class to construct the overall genetic proxies for drug effects: Insulin (*INSR*), Metformin (*GPD1*), Sulfonylureas (*ABCC8/KCNJ11* + *ABCB11* + *CPT1A* + *KCNJ11* + *VEGFA*), TZDs (*ESRRA* + *SERPINE1* + *SLC29A1* + *PPARD*), GLP-1RA (*GLP1R*), and SGLT2i (*SLC5A2* + *SLC5A1*). All drug-level instruments were validated by positive control analyses.

### Effects of genetic variation in glucose-lowering drug targets on CKM syndrome risk by MR and meta-analysis

The associations of genetically proxied glucose-lowering drug targets with CKM syndrome-related diseases across three independent GWAS datasets for each trait were evaluated by MR **(Table S7)** and the pooled estimates from meta-analysis were regarded as the final causal effect estimates **(Table S8, Figure S1-S14)**.

Based on HbA1c-derived IVs, *GPD1* (Metformin) significantly reduced CAD risk (OR = 0.51, 95% CI 0.40-0.66, *P* < 0.001, FDR < 0.001), alongside a potential benefit for PAD (OR = 0.99, 95% CI 0.99-1.00, *P* = 0.035). For Sulfonylureas, *ABCC8/KCNJ11* showed suggestive protective associations against VTE (OR = 0.98, 95% CI 0.96-1.00, *P* = 0.039) and MetS (OR = 0.58, 95% CI 0.38-0.88, *P* = 0.011). *ABCB11* demonstrated significant protective effects against CAD (OR = 0.81, 95% CI 0.76-0.86, *P* < 0.001, FDR < 0.001) and MetS (OR = 0.80, 95% CI 0.74-0.85, *P* < 0.001, FDR < 0.001), with a suggestive benefit for CKD (OR = 0.89, 95% CI 0.81-0.97,* P* = 0.009). *CPT1A* was significantly associated with a lower risk of MetS (OR = 0.18, 95% CI 0.09-0.37, *P* < 0.001, FDR < 0.001). Among TZD targets, *ESRRA* conferred significant reductions in the risks of CAD (OR = 0.30, 95% CI 0.21-0.41, *P* < 0.001, FDR < 0.001), AF (OR = 0.98, 95% CI 0.96-0.99, *P* < 0.001, FDR = 0.002), and MetS (OR = 0.15, 95% CI 0.10-0.24, *P* < 0.001, FDR < 0.001), whereas *SERPINE1* showed suggestive associations with increased risks of CKD (OR = 3.91, 95% CI 1.08-14.11, *P* = 0.037) and MetS (OR = 3.63, 95% CI 1.19-11.07, *P* = 0.023). *SLC29A1* was significantly protective against CAD (OR = 0.41, 95% CI 0.27-0.63, *P* < 0.001, FDR < 0.001) but increased the risks of HF (OR = 1.48, 95% CI 1.15-1.91,* P* = 0.002, FDR = 0.016) and AF (OR = 1.02, 95% CI 1.01-1.02, *P* < 0.001, FDR = 0.002). For SGLT2i, *SLC5A1* showed a suggestive association with lower VTE risk (OR = 0.98, 95% CI 0.97-1.00, *P* = 0.019), whereas *SLC5A2* demonstrated significant protection against HF (OR = 0.70, 95% CI 0.57-0.86, *P* = 0.001, FDR = 0.005) and MetS (OR = 0.44, 95% CI 0.32-0.60, *P* < 0.001, FDR < 0.001).

Gene expression-based analyses further supported these findings.* INSR* (Insulin) was suggestively associated with increased MetS risk (OR = 1.07, 95% CI 1.02-1.13, *P* = 0.013). For Sulfonylureas, *KCNJ11* showed suggestive protective associations with HF (OR = 0.94, 95% CI 0.90-0.99, *P* = 0.010), CKD (OR = 0.94, 95% CI 0.88-1.00, *P* = 0.048), and MetS (OR *=* 0.94, 95% CI 0.90-0.99, *P* = 0.031), while *VEGFA* was suggestively associated with reduced CAD risk (OR = 0.98, 95% CI 0.96-1.00, *P* = 0.025). Among TZDs, *PPARD* demonstrated a suggestive association with stroke (OR = 0.99, 95% CI 0.99-1.00, *P* = 0.045) and an increased HF risk (OR = 1.10, 95% CI 1.03-1.18, *P* = 0.006). For GLP-1RA, *GLP1R* exhibited robust protective associations with CAD (OR = 0.90, 95% CI 0.84-0.96, *P* = 0.002, FDR = 0.043) and CKD (OR = 0.87, 95% CI 0.79-0.95, *P* = 0.002, FDR = 0.043), alongside a suggestive protective effect against HF (OR = 0.92, 95% CI 0.86-0.99, *P* = 0.020).

**Figure 2:**
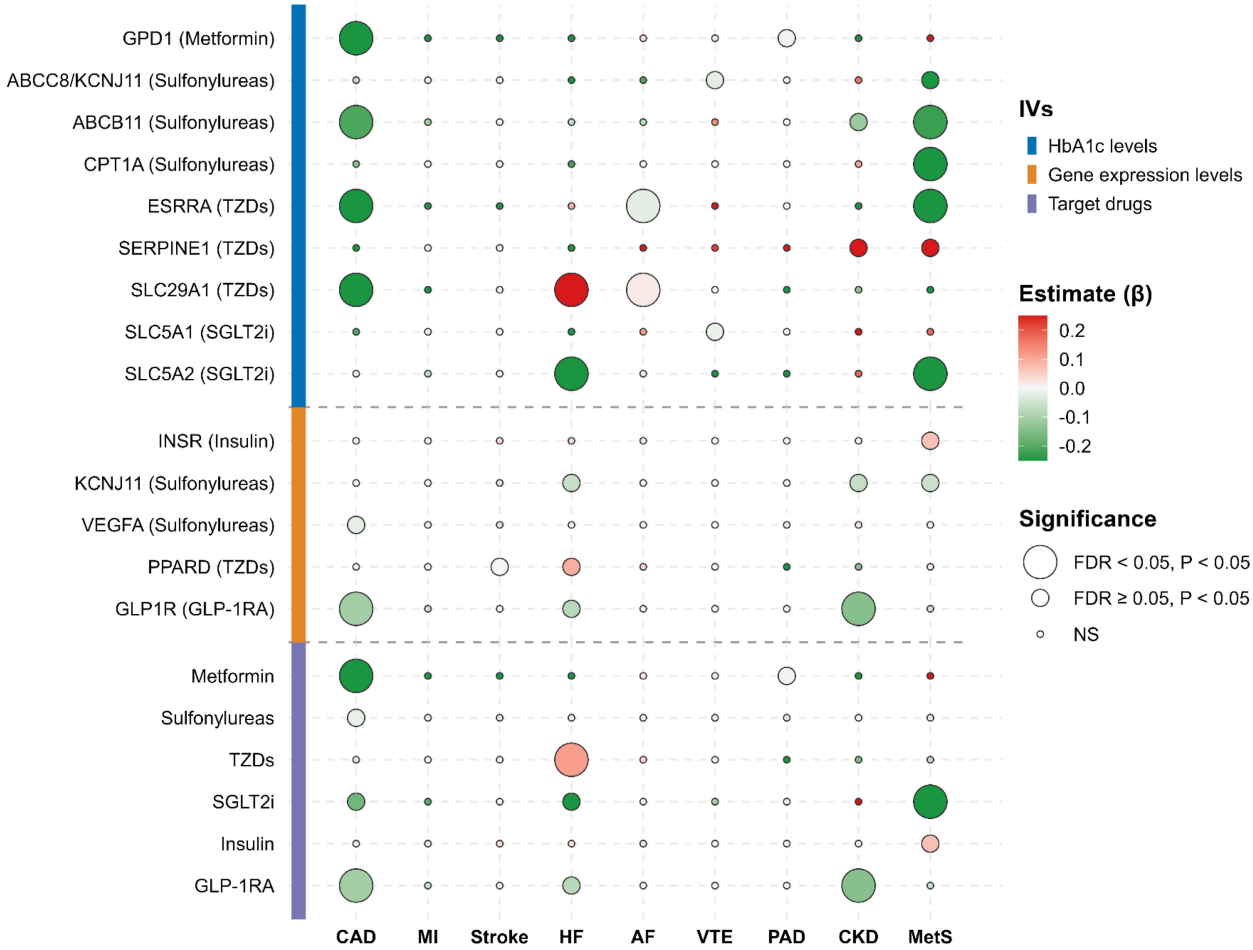
** MR and meta-analysis of glucose-lowering drug targets and CKM syndrome-related diseases.** The bubble heatmap illustrates the association between genetically proxied drug targets and nine CKM syndrome outcomes. Targets are categorized by IV sources: HbA1c levels, gene expression levels, and overall target drugs. A red bubble indicates a positive association, and a green bubble indicates a negative association. Bubble size corresponds to the level of statistical significance (large for FDR < 0.05, medium for *P* < 0.05, and small for non-significant results).

At the drug overall level, pooled analyses revealed that Metformin significantly reduced the risk of CAD (OR = 0.51, 95% CI 0.40-0.66, *P* < 0.001, FDR < 0.001) and suggestively reduced PAD risk (OR = 0.99, 95% CI 0.99-1.00, *P* = 0.035). Sulfonylureas were suggestively associated with lower CAD risk (OR = 0.98, 95% CI 0.96-1.00, *P* = 0.024). TZDs significantly increased HF risk (OR = 1.12, 95% CI 1.05-1.20, *P* < 0.001, FDR = 0.007). SGLT2i significantly reduced the risk of MetS (OR = 0.64, 95% CI 0.50-0.82, *P* < 0.001, FDR = 0.007), with suggestive reductions in CAD (OR = 0.85, 95% CI 0.73-0.98, *P* = 0.027) and HF (OR = 0.66, 95% CI 0.44-0.98, *P* = 0.040). Insulin suggestively increased the risk of MetS (OR = 1.07, 95% CI 1.02-1.13, *P* = 0.013). Finally, GLP-1RA conferred significant protection against CAD (OR = 0.90, 95% CI 0.84-0.96, *P* = 0.002, FDR = 0.021) and CKD (OR = 0.87, 95% CI 0.79-0.95, *P* = 0.002, FDR = 0.021), and suggestive protection against HF (OR = 0.92, 95% CI 0.86-0.99, *P* = 0.020). These results are summarized in **Figure 2**.

### Summary-based MR

**Figure 3:**
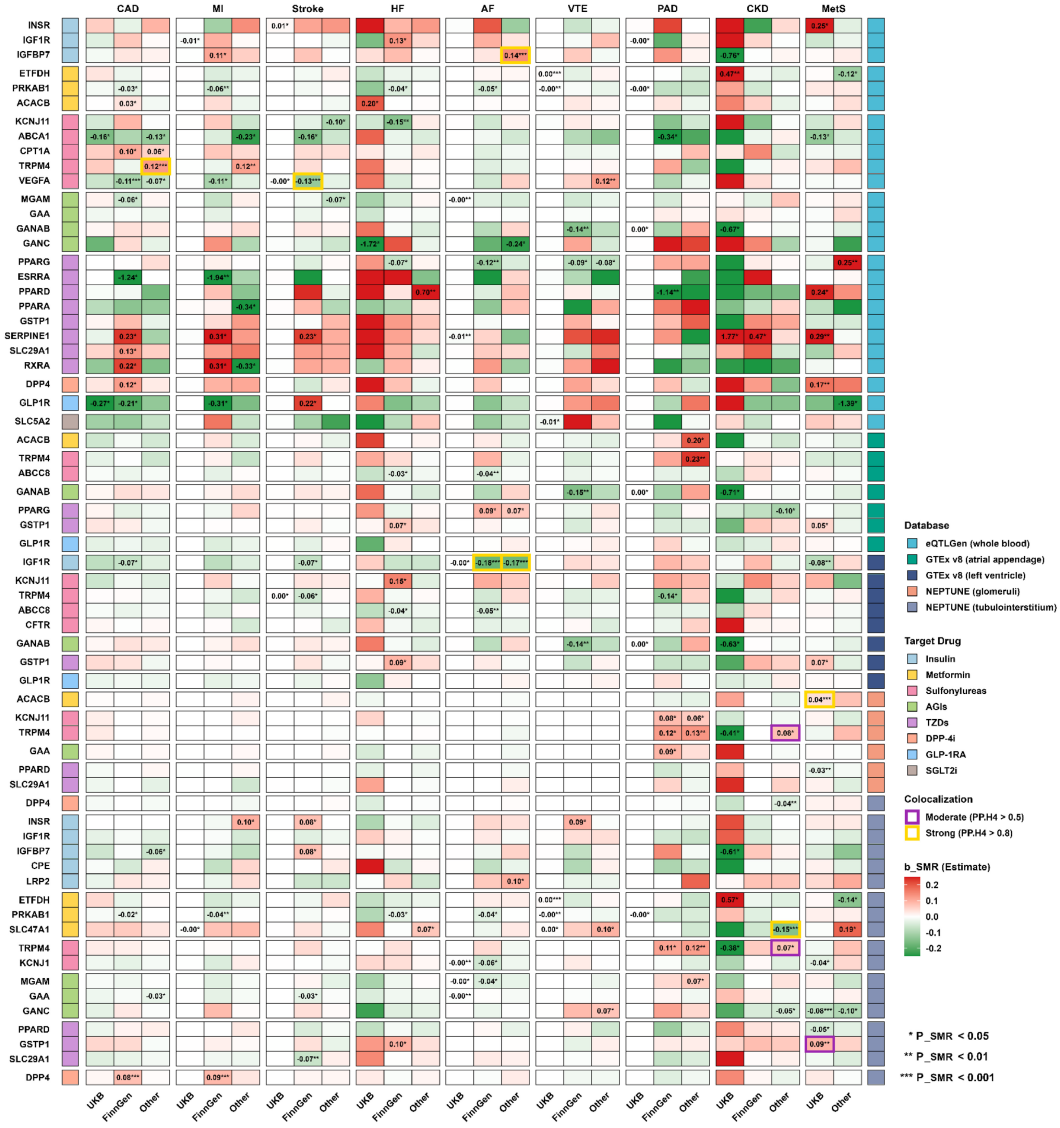
** SMR and colocalization analysis of glucose-lowering drug targets and CKM syndrome-related diseases.** The matrix heatmap illustrates the association between the genetically predicted expression of drug target genes across five eQTL databases and nine CKM syndrome outcomes. A red region indicates a positive association, a green region indicates a negative association, and the β_SMR estimates are displayed if the results are significant (*P*_SMR < 0.05). Asterisks denote the level of significance (* *P*_SMR < 0.05, ** *P*_SMR < 0.01, *** *P*_SMR < 0.001). Additionally, highlighted cell borders denote the posterior probability of colocalization, with gold and purple borders representing strong (PP.H4 > 0.8) and moderate (PP.H4 > 0.5) evidence, respectively.

In the SMR analyses, several drug targets previously identified in our MR analyses were further validated **(Figure 3, Table S9)**. Specifically, in whole blood, genetically proxied activation of *GLP1R* was validated as protective against CAD (OR = 0.81, 95% CI 0.66-0.99, *P*_SMR = 0.034, *P*_HEIDI = 0.151). Similarly, for Sulfonylureas, the genetically proxied inhibition of *KCNJ11* was confirmed to reduce HF risk (OR = 0.86, 95% CI 0.77-0.96, *P*_SMR = 0.009, *P*_HEIDI = 0.754), and *VEGFA* was validated as protective against CAD (OR = 0.90, 95% CI 0.84-0.96, *P*_SMR = 0.001, *P*_HEIDI = 0.086). In contrast, *INSR* (Insulin) was verified to increase MetS risk (OR = 1.28, 95% CI 1.06-1.55, *P*_SMR = 0.011, *P*_HEIDI = 0.146). Among TZD targets, genetically proxied inhibition of *SERPINE1* showed consistent harmful associations, with increased risks of CKD across two independent datasets (OR = 5.88, 95% CI 1.09-31.67, *P*_SMR = 0.039, *P*_HEIDI = 0.497; and OR = 1.60, 95% CI 1.08-2.39, *P*_SMR = 0.021, *P*_HEIDI = 0.321), as well as an increased MetS risk (OR = 1.33, 95% CI 1.10-1.61, *P*_SMR = 0.003, *P*_HEIDI = 0.767). Furthermore, SMR analyses utilizing eQTLs from cardiac and renal tissues provided complementary insights. Notably, for Sulfonylureas, the genetically proxied inhibition of *ABCC8* in the atrial appendage and left ventricle was associated with lower risks of AF and HF. Additionally, the genetically proxied activation of *PRKAB1* in whole blood and renal tubulointerstitium exhibited broad protective effects against multiple cardiovascular diseases, such as CAD, AF, HF, MI, PAD, and VTE (all *P*_SMR < 0.05, *P*_HEIDI > 0.05).

### Colocalization analysis

Consistent with SMR analyses (**Figure 3**), strong evidence for a shared causal variant was observed for left ventricle *IGF1R* eQTL and AF across two independent datasets (PPH4 = 0.990 and 0.968) (**Table S10**). High posterior probabilities were also identified for whole blood *TRPM4, IGFBP7, VEGFA* eQTL with CAD (PPH4 = 0.932), AF (PPH4 = 0.825) and stroke (PPH4 = 0.812), respectively. Meanwhile, moderate evidence of colocalization was observed for *GSTP1* eQTL and MetS risk in tubulointerstitium (PPH4 = 0.591). However, no strong evidence for colocalization was observed for the causal associations identified through the two-sample MR analyses.

## Discussion

This is the first and most comprehensive MR study to date investigating the potential benefits and risks of genetic variation in glucose-lowering drug targets on CKM syndrome-related diseases. Using multi-dataset two-sample MR and meta-analysis, genetically proxied SGLT2i and GLP-1RA demonstrated prominent and consistent protective associations across three comorbid conditions, while metformin and sulfonylureas showed moderate benefits in one to two comorbid conditions. In contrast, insulin and specific targets of TZDs were associated with adverse outcomes. Additionally, supplementary validation through SMR identified several key target genes mediating these drug-disease associations. From a genetic perspective, these findings offer new insights into the therapeutic potential of glucose-lowering drugs in the context of CKM syndrome comorbidities.

SGLT2i lower blood glucose levels by reducing the reabsorption of sodium and glucose in the proximal tubules of the kidney. They also exert multiple beneficial effects, including weight loss and improved lipid metabolism, and have been shown to significantly improve various aspects of MetS, such as obesity, dyslipidemia, and insulin resistance 39-40. In recent years, SGLT2i have garnered widespread attention due to their ability to reduce cardiovascular mortality, heart failure hospitalization rates, and the incidence of adverse kidney outcomes 41-43. These benefits extend far beyond glucose control and may be associated with antioxidant effects, reduced inflammation and fibrosis, decreased circulatory load, enhanced erythropoiesis, modulation of mitochondrial function, and improved cardiac energy metabolism and vascular function 44-46. Notably, the favorable effects on the heart and kidneys appear to be independent of glucose-lowering effects, as similar phenomena have been observed in individuals without diabetes 47-48. Our MR study, focusing on the target-specific perturbation of SGLT2i, rather than generalized glucose-lowering effects, aligns with prior evidence, suggesting that SGLT2i are associated with a reduced risk of CAD, HF, and MetS. However, we did not observe a potential benefit of SGLT2i on CKD, which may be attributed to the limitations of the data used, which did not adequately cover the diversity of different CKD subtypes or patient populations. The benefits of SGLT2i may be more relevant to specific CKD subtypes [e.g., diabetic kidney disease]. Additionally, we noted that SGLT2i may have a protective effect on VTE through the *SLC5A1* gene, which could provide new perspectives for the drug's indications. Indirect evidence suggests that SGLT2i might reduce the risk of ischemic stroke in patients with AF and diabetes, with its potential mechanism of preventing thrombosis related to the improvement of inflammatory states in AF patients 49. A nationwide retrospective cohort study from China indicated that, compared to DPP-4 inhibitors, SGLT2i are associated with a lower risk of VTE, and this finding was further supported by a subsequent meta-analysis 50.

GLP-1RA were initially developed as glucose-lowering drugs by stimulating insulin secretion, inhibiting glucagon release, and slowing gastric emptying 51. However, large RCTs have revealed an unexpected benefit: GLP-1RA acts as a systemic metabolic modulator, providing cardiovascular and renal benefits far beyond glucose control and weight loss. A recent meta-analysis, which included 67,769 diabetic patients across 11 RCTs, showed that, compared to a placebo, GLP-1RA reduced the risk of composite renal adverse events by 18%, the risk of renal failure by 16%, and the risk of major adverse cardiovascular events by 13% 52. Similarly, the ERA Diabesity working group reviewed 9 high-quality RCTs and 4 real-world cohorts (with a total sample size of over 170,000 and a maximum follow-up of 8.5 years), reporting a 14% reduction in the overall risk of MACE, a 31% reduction in heart failure hospitalization rates in high-risk subgroups, and a 22% reduction in the risk of adverse renal outcomes 53. Based on *GLP1R* expression-based MR analysis independent of HbA1c, clear protective associations of genetically proxied GLP-1RA with CAD, HF, and CKD were observed, with the effect on CAD further validated in SMR analysis. Mechanistically, the beneficial effects of GLP-1RA on the heart and kidneys may be related to its suppression of oxidative stress, reduction of inflammation and fibrosis 54-59. Specifically, activation of *GLP1R* reduces the production of reactive oxygen species (ROS) through the downregulation of HO-1 and non-receptor-mediated NADPH oxidase, and inhibits the activity of NF-κB, thus reducing the production of pro-inflammatory chemokines, cytokines, adhesion molecules, and pro-fibrotic factors 60. Studies have shown that the most common causes of death in DKD patients are heart failure and CAD, further highlighting the potential of GLP-1RA as a therapeutic strategy for managing comorbid conditions 61.

Metformin has been used for over 70 years in the treatment of diabetes 62. It controls blood glucose levels by inhibiting hepatic gluconeogenesis, enhancing peripheral insulin sensitivity, and reducing intestinal glucose absorption. It remains one of the most commonly prescribed drugs for the treatment of T2D. In this study, we observed potential benefits of metformin for CAD and PAD. A substantial body of evidence indicates that metformin significantly reduces the risk of major cardiovascular events and mortality in patients with ASCVD associated with T2D 63-68. Although previous studies have suggested metformin's significant potential in combating atherosclerosis, its precise mechanisms remain unclear. The largest and longest double-blind placebo-controlled RCT, the REmoval trial (REversing with MetfOrmin Vascular Adverse Lesions), found that metformin significantly slowed the progression of atherosclerosis in patients with type 1 diabetes, as measured by the averaged maximal carotid intima-media thickness (-0.013 mm/year, -0.024 to -0.003; *P* = 0.0093) 69. Li et al. further confirmed in a cholesterol-fed rabbit model of atherosclerosis that metformin intervention significantly reduced atherosclerotic plaque formation and inhibited the phosphorylation of IκB and activation of NF-κB in the vascular wall, while lowering serum hs-CRP levels 70. Similarly, Forouzandeh et al. observed similar results in ApoE^-/-^ C57BL/6J mice, with the protective effects potentially attributed in part to the downregulation of the angiotensin II type 1 receptor 71. Additional studies on metformin's potential mechanisms in combating atherosclerosis have shown that it may enhance endothelial function through the activation of the AMP-activated protein kinase (AMPK) pathway to promote nitric oxide (NO) production 72-73. Consistent with this, our SMR analysis confirmed that genetically predicted *PRKAB1* (encoding the AMPK β1 subunit) exhibited broad protective effects across multiple cardiovascular outcomes. Furthermore, metformin may inhibit the abnormal migration of vascular smooth muscle cells and delay intimal thickening 74; regulate triglyceride levels and HDL functionality 75-76; and suppress the activation of NF-κB in endothelial cells and vascular smooth muscle cells, thereby reducing the secretion of pro-inflammatory cytokines induced by interleukin-1β 77, which weakens the chronic inflammation process that contributes to atherosclerosis 78. Other studies have reported that metformin can prevent local complications in PAD patients 79 and improve survival rates 80. Additionally, the ongoing MOBILE IC trial (NCT05132439) aims to evaluate the ability of metformin to improve function in patients with intermittent claudication and delay the progression of PAD, which could provide robust evidence for positioning metformin as a novel therapeutic strategy for PAD management 81.

As insulin secretagogues, sulfonylureas have traditionally been used for blood glucose control, but their role in cardioprotective and nephroprotective outcomes remains a subject of ongoing debate 82-85. In recent years, with the emergence of newer glucose-lowering drugs such as SGLT2i and GLP-1RA, research on the cardiovascular and renal safety of sulfonylureas often seems to take a backseat, with conflicting conclusions. A large-scale meta-analysis (47 RCTs, 37,650 patients) found that sulfonylureas did not significantly increase the risk of all-cause mortality, cardiovascular mortality, MI, or stroke 86. However, some high-quality observational studies have shown that sulfonylureas are associated with an increased risk of cardiovascular events and mortality, particularly when compared with drugs like metformin 87. Despite this, our MR-meta analysis indicates that certain targets of sulfonylureas, such as *ABCC8/KCNJ11*, *ABCB11*, and *VEGFA*, are significantly associated with a reduction in the risk of diseases like CAD, CKD, and MetS. At the drug level, sulfonylureas are also linked to a reduction in CAD risk. However, given the lack of definitive evidence in real-world settings, our conclusions must be interpreted with caution.

We also identified some adverse effects associated with certain drugs. *INSR*, a key target gene in the insulin signaling pathway, is typically linked to insulin resistance and other manifestations of MetS 88. However, our study found that the genetic proxy for insulin, which activates *INSR*, while lowering blood glucose, also increases the risk of MetS. This can be explained by several factors: On one hand, insulin, as a potent anabolic hormone, promotes fat synthesis and storage, leading to obesity 89-90. On the other hand, excessive insulin use increases the risk of hypoglycemia, triggering the release of counter-regulatory hormones that promote lipolysis, increase free fatty acids, and induce insulin resistance, ultimately exacerbating the metabolic abnormalities associated with MetS 91-92. It is important to emphasize that MR estimates reflect the lifetime, moderate disruption of the target, and the effect size should not be directly equated to the results observed in short-term clinical trials. While insulin is effective in lowering blood glucose, its long-term use may have potential adverse effects on overall metabolic health. Furthermore, TZDs, as classic insulin sensitizers, improve insulin resistance and effectively lower blood glucose by activating the PPARγ pathway. However, their cardiovascular and metabolic effects have been a subject of debate. In our MR study, the effects of TZD-related targets were complex and inconsistent: for instance, *ESRRA* was associated with reduced risks of CAD, AF, and MetS, while targets such as *SERPINE1*, *SLC29A1*, and *PPARD* were linked to adverse outcomes, including HF, AF, CKD, and MetS. Overall, the drug-level results indicated a significant increase in the risk of HF associated with TZDs, a finding well-supported by numerous real-world studies 93-97. The main mechanisms for this are likely fluid retention, weight gain, and enhanced sodium and water reabsorption, leading to increased cardiac preload and deterioration of heart function 98-99.

This study has several limitations. First, the genetic data used in this research is solely derived from individuals of European descent, which may limit the generalizability of our findings. Second, our genetic instruments capture only highly specific targeted effects, without accounting for potential off-target effects. Third, MR estimates reflect lifelong, low-intensity genetic modulation of drug targets, whereas clinical pharmacotherapy typically involves short-term, high-intensity drug exposure in middle-aged or elderly patients. Therefore, the absolute effect sizes should not be directly equated with the anticipated magnitude of benefit in real-world clinical settings, nor should they be interpreted as clinical recommendations for patient management. Fourth, although we employed various methods to ensure the robustness of the analysis, the inherent potential for pleiotropy and bias in MR analyses remains unavoidable. Despite selecting genetic variants from the cis-regions of each diabetes-related target gene, some variants may still influence disease risk through other pathways. Specifically, results lacking strong colocalization support should be interpreted with caution, as they may merely represent pleiotropic or proxy associations rather than definitive drug-target effects. Further multi-exposure MR analysis could help deepen the validation of these causal relationships. Fifth, as this study fundamentally relies on robust SNPs derived from large-scale GWAS and tissue-specific eQTL data, not all drug targets possessed available IVs to be comprehensively explored. Additionally, we did not explore the effects in specific disease subtypes, such as the treatment responses in patients with DKD. Finally, while MR provides strong causal inference evidence, the results still need to be validated through larger-scale clinical and mechanistic trials.

This is the first and most comprehensive systematic evaluation of the genetic variations in glucose-lowering drug targets and their potential benefits and risks in diseases related to CKM syndrome. From a genetic perspective, our findings provide mechanistic evidence supporting the broad cardiorenal benefits of SGLT2i and GLP-1R targets, while highlighting potential genetic liabilities associated with insulin and specific TZD targets. While not serving as direct clinical guidelines, these findings have significant implications for future target validation, drug repurposing, and the development of personalized precision therapies.

## Supplementary Material

Supplementary figures.

Supplementary tables.

## Data Availability

All data used in this study are publicly available as described in **Table S4**. Summary statistics can be accessed via specified repositories.
