# Supplementary material for: Genetic Evidence for the Benefits and Risks of Glucose-Lowering Drugs on Cardiovascular-Kidney-Metabolic Syndrome: A Drug-Target Mendelian Randomization Study
Source: Int J Med Sci. 2026 May 11;23(7):2197–208. doi: 10.7150/ijms.133077 (PMC13280743; doi:10.7150/ijms.133077)
Supplement: Supplementary file 1 — Supplementary figures. [file ijmsv23p2197s1.pdf]

Supplementary Figures

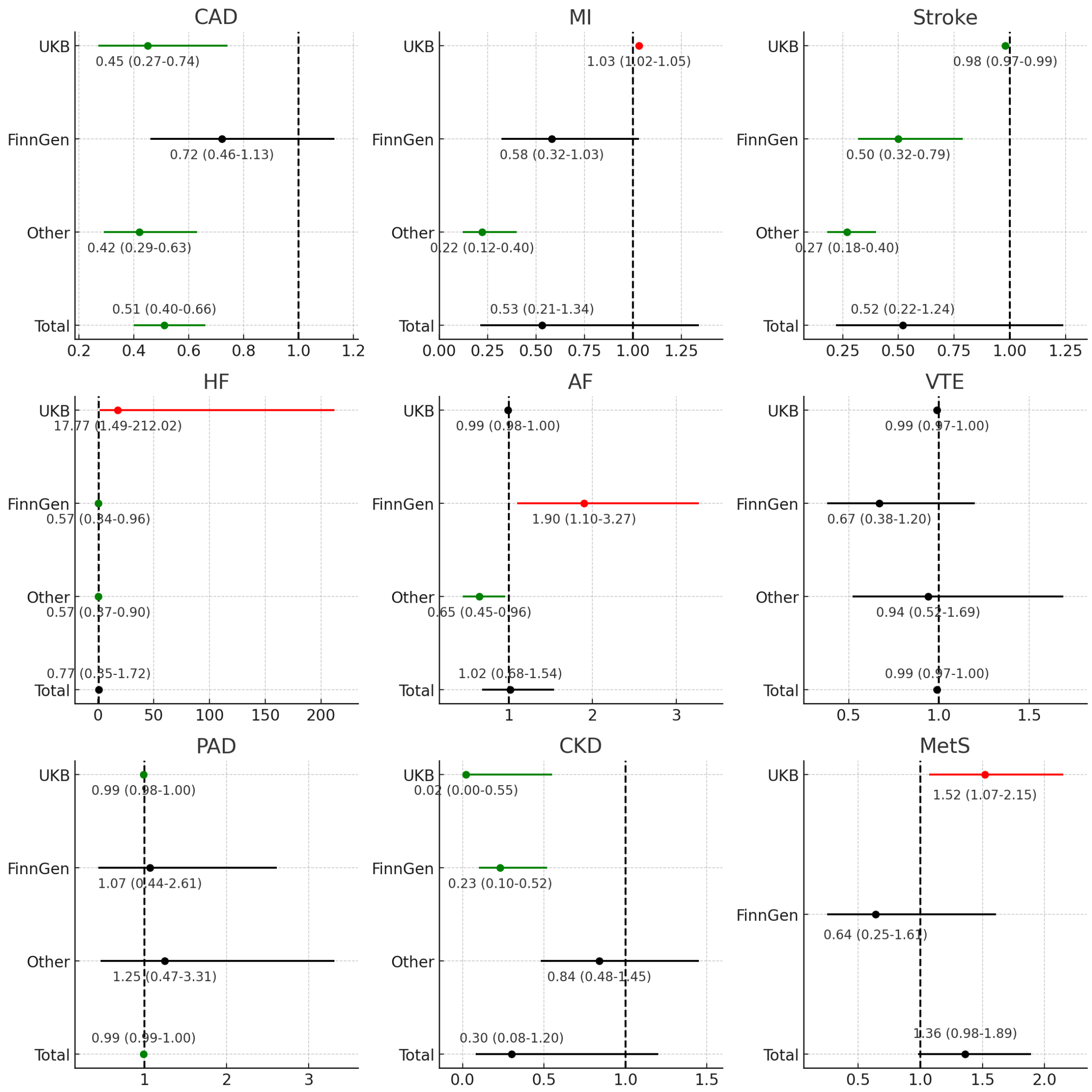

Figure S1. Pooled MR estimates of GPD1 (Metformin) on nine CKM syndrome－related diseases.

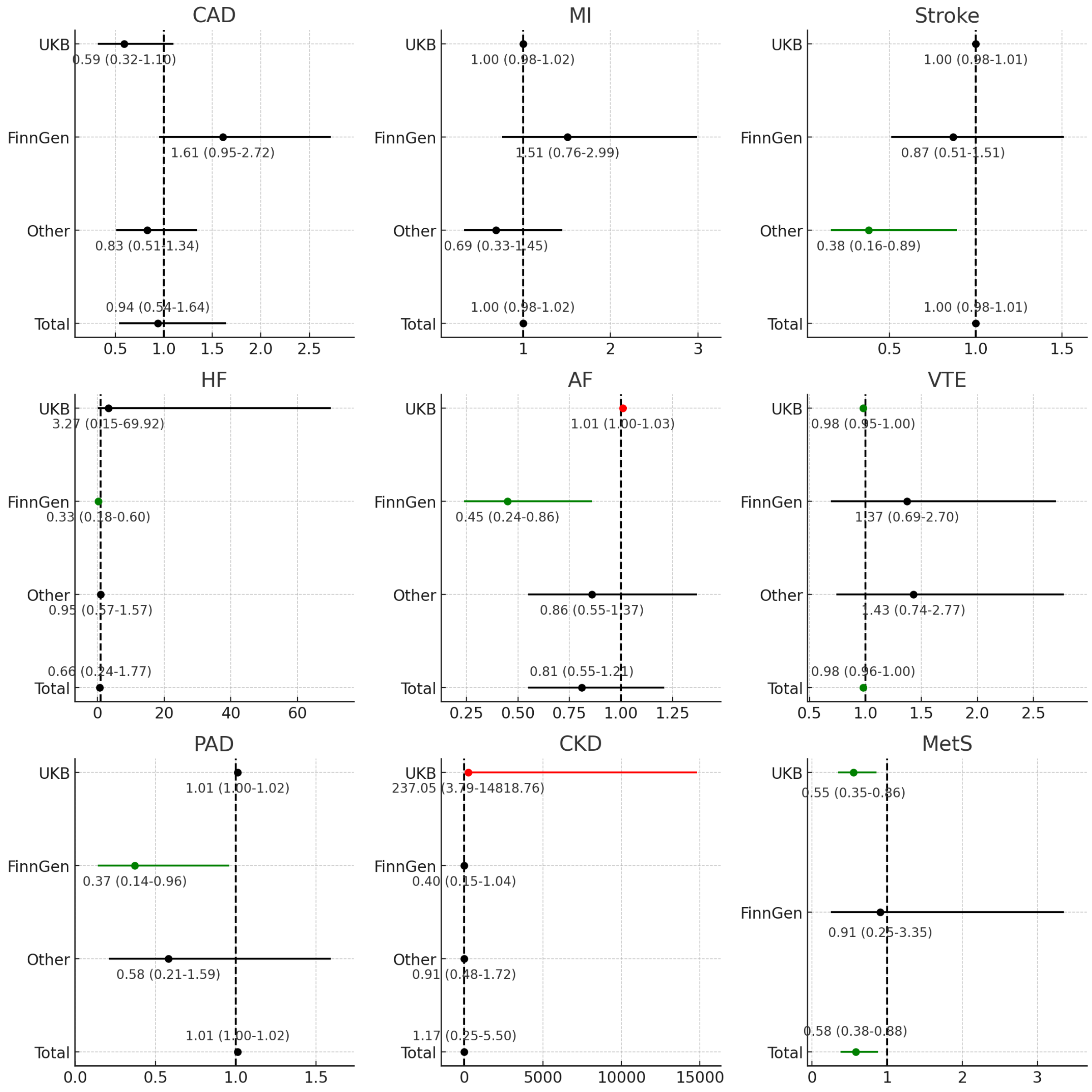

Figure S2. Pooled MR estimates of ABCC8/KCNJ11 (Sulfonylureas) on nine CKM syndrome－related diseases.

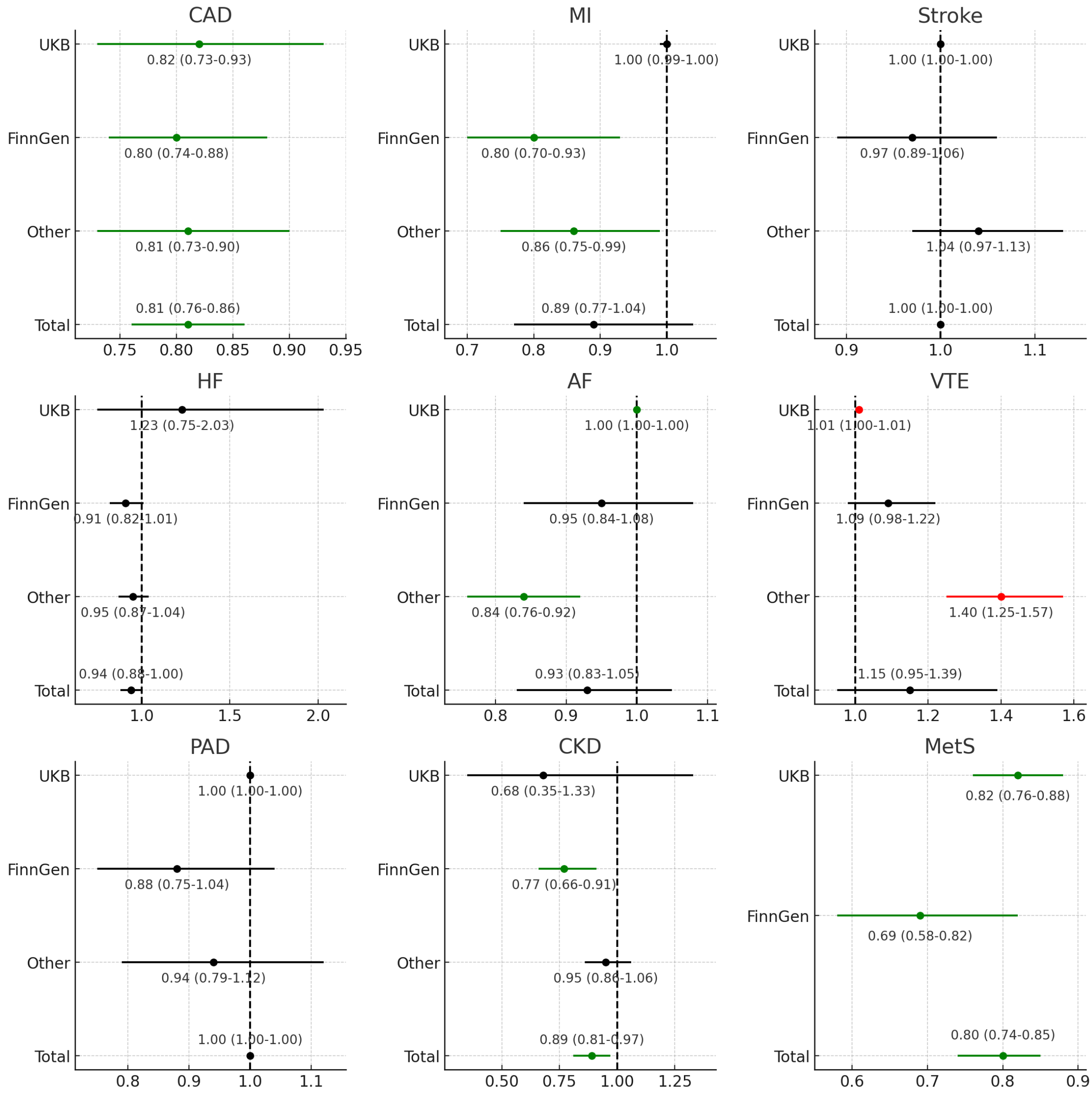

Figure S3. Pooled MR estimates of ABCB11 (Sulfonylureas) on nine CKM syndrome – related diseases.

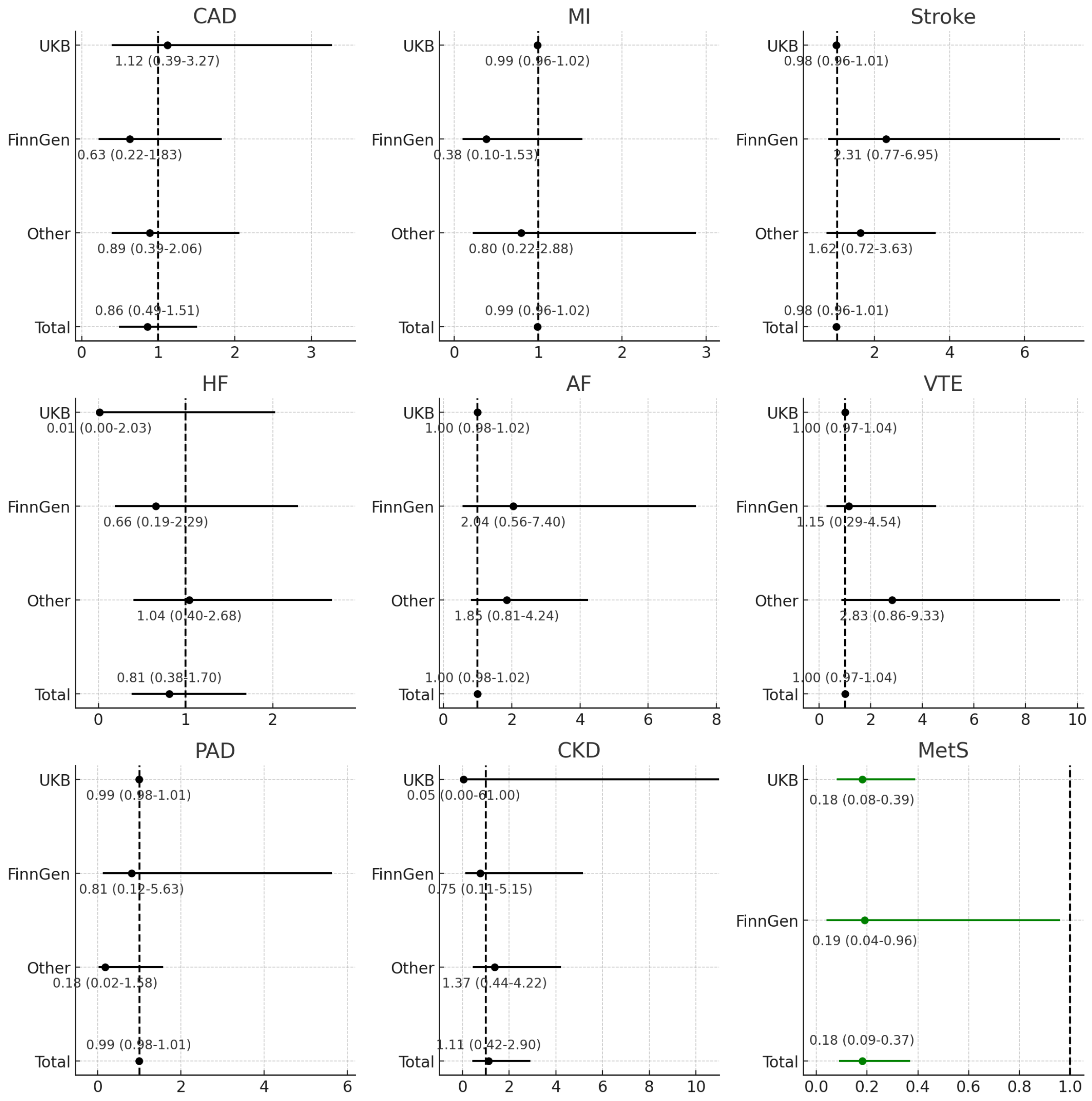

Figure S4. Pooled MR estimates of CPT1A (Sulfonylureas) on nine CKM syndrome – related diseases.

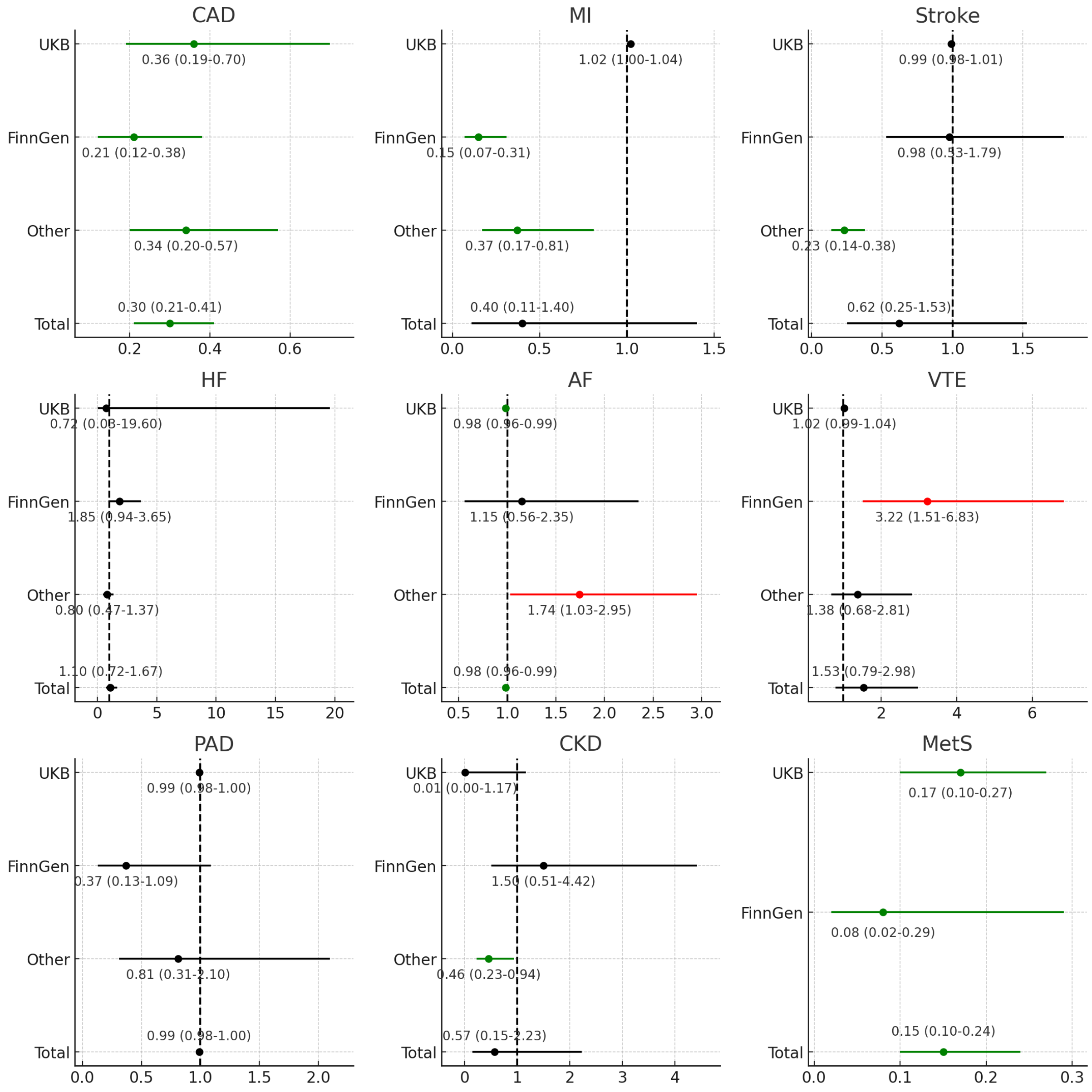

Figure S5. Pooled MR estimates of ESRRA (TZDs) on nine CKM syndrome‑related diseases.

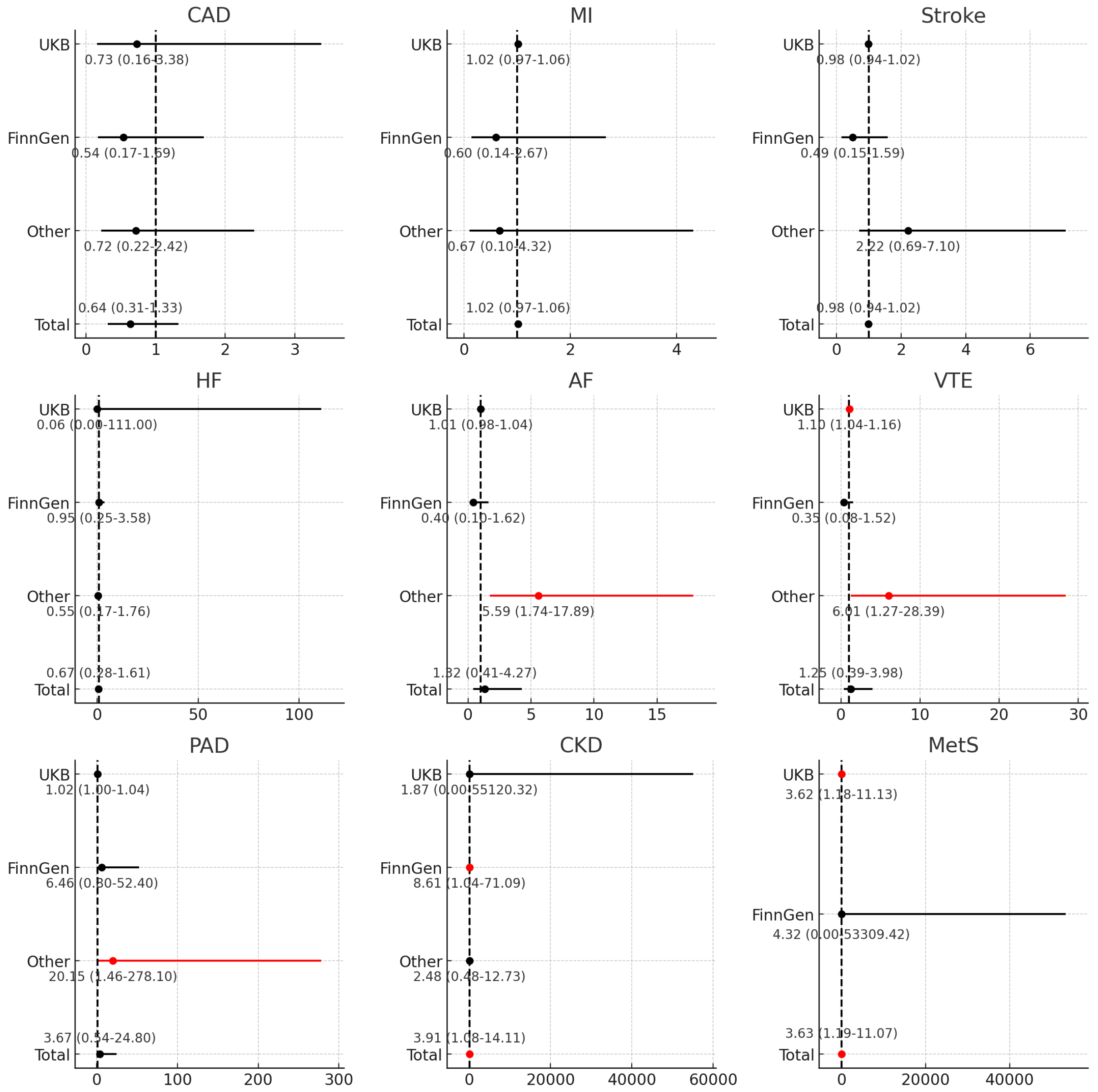

Figure S6. Pooled MR estimates of SERPINE1 (TZDs) on nine CKM syndrome－related diseases.

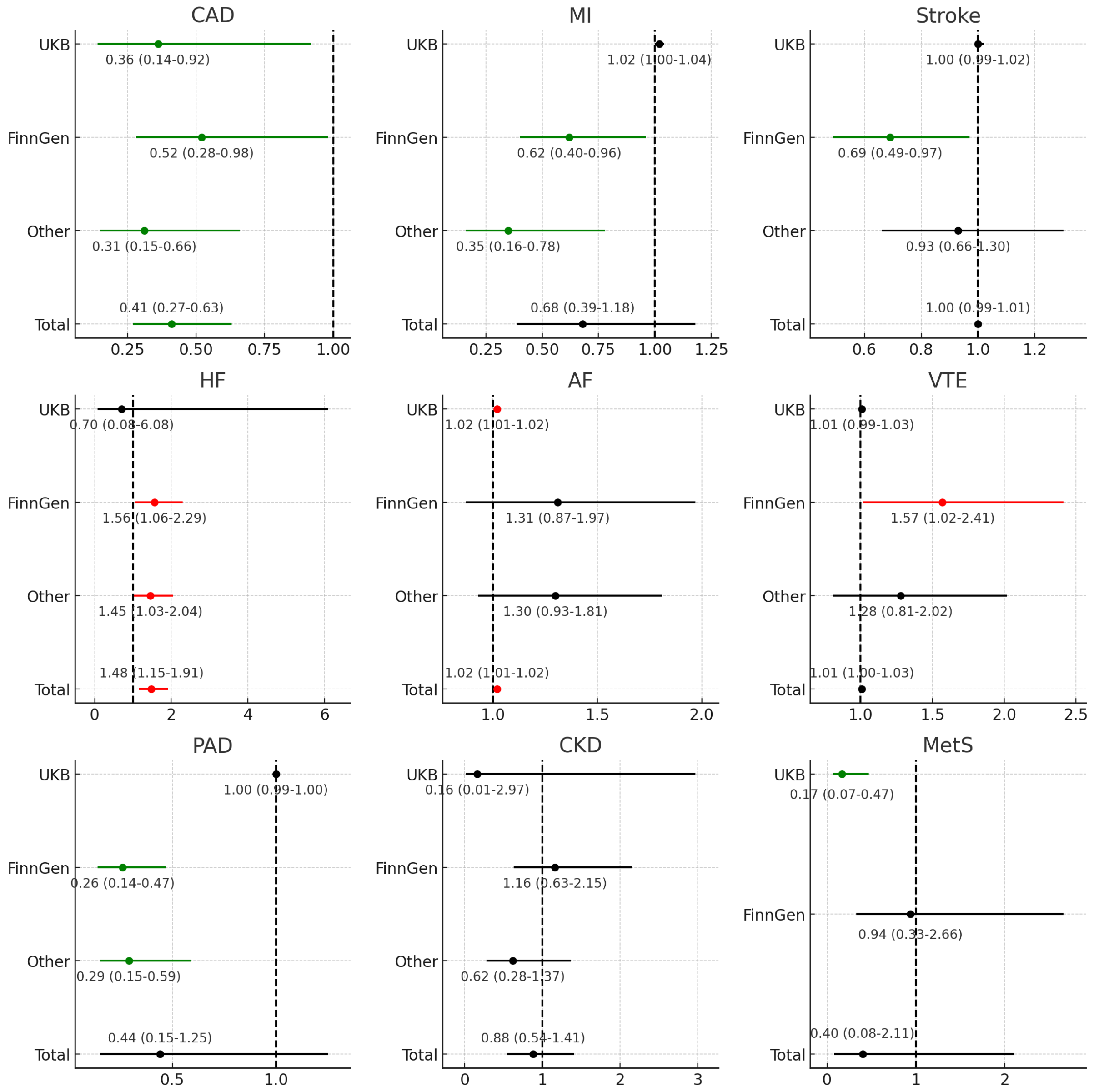

Figure S7. Pooled MR estimates of SLC29A1 (TZDs) on nine CKM syndrome - related diseases.

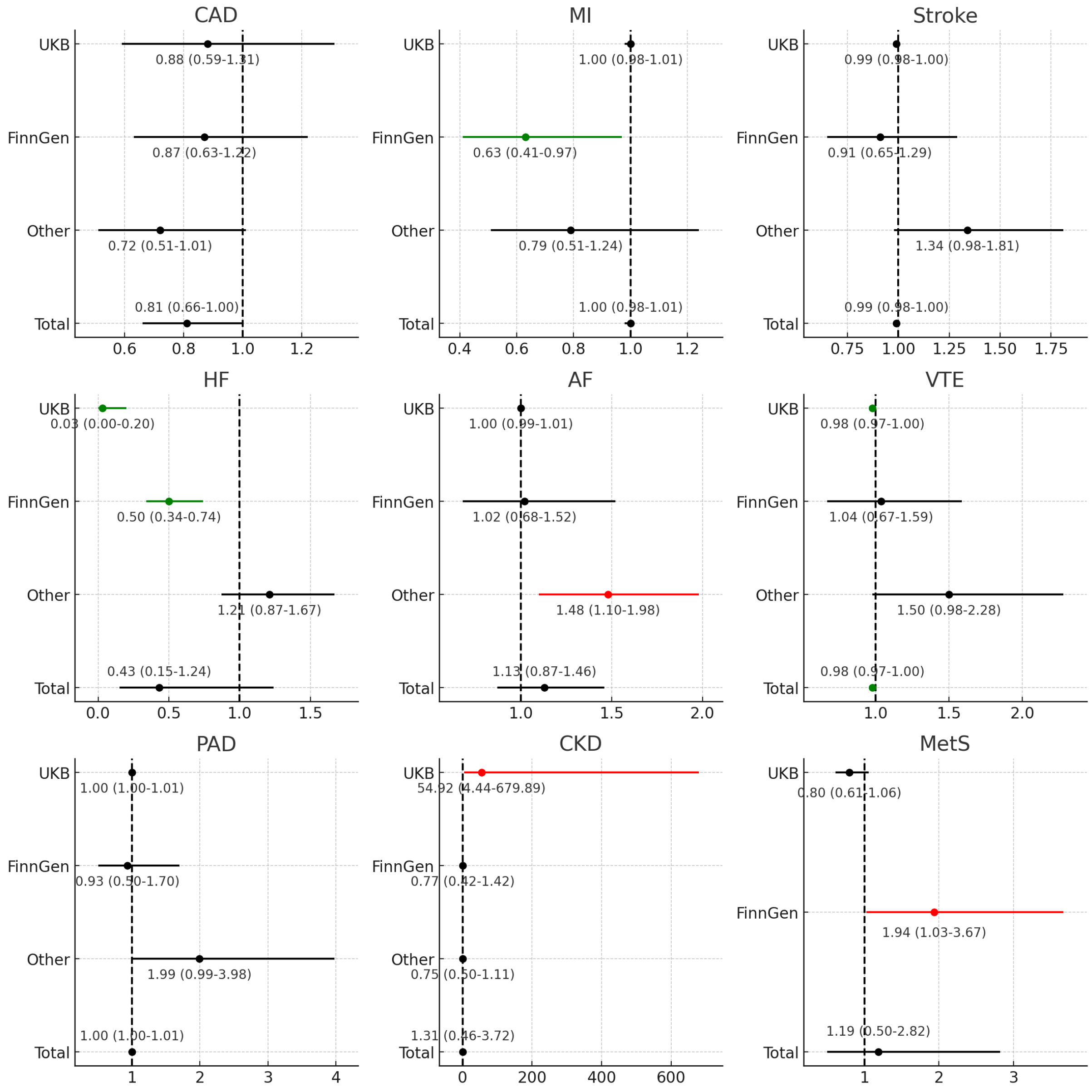

Figure S8. Pooled MR estimates of SLC5A1 (SGLT2i) on nine CKM syndrome-related diseases.

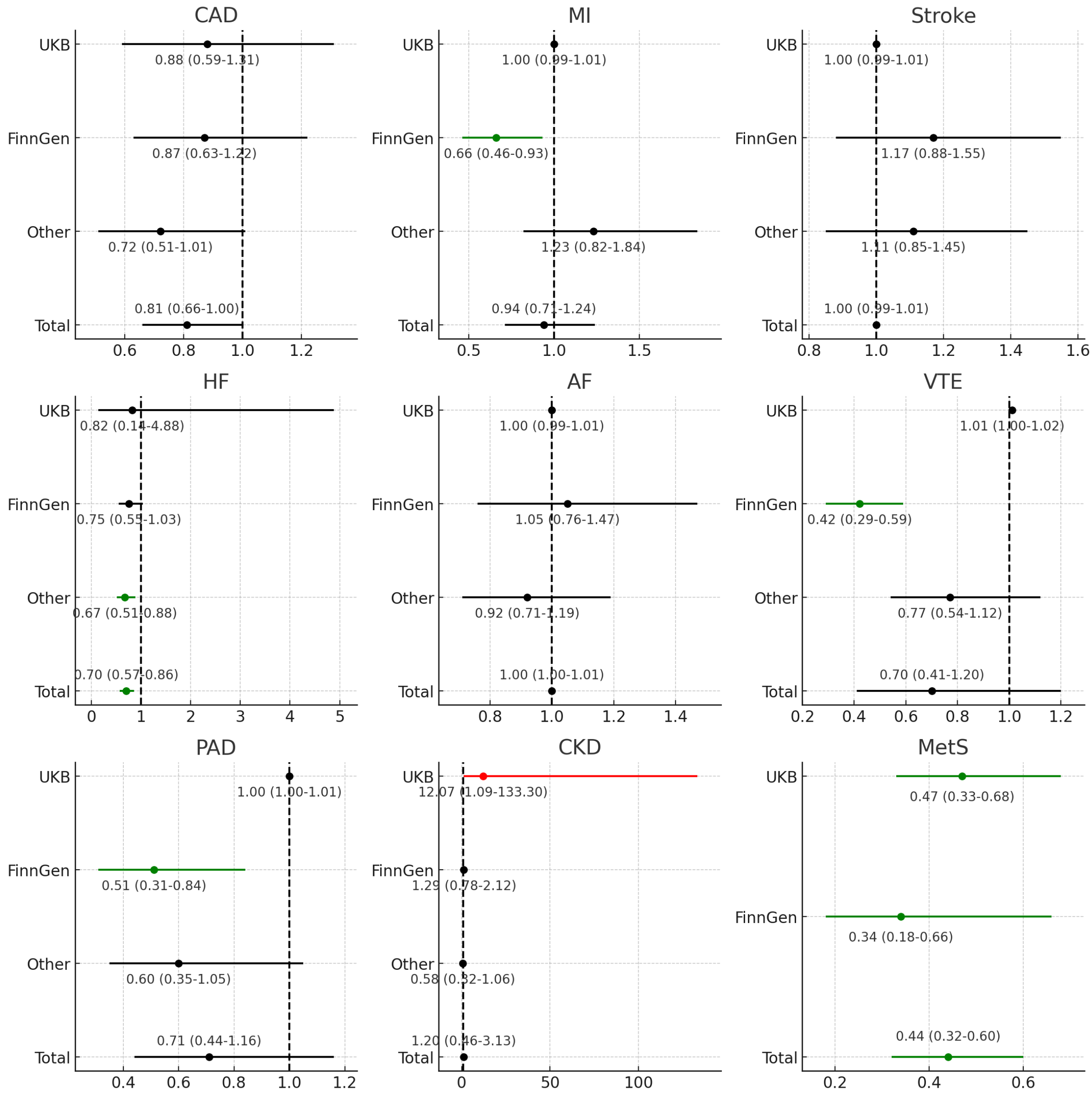

Figure S9. Pooled MR estimates of SLC5A2 (SGLT2i) on nine CKM syndrome－related diseases.

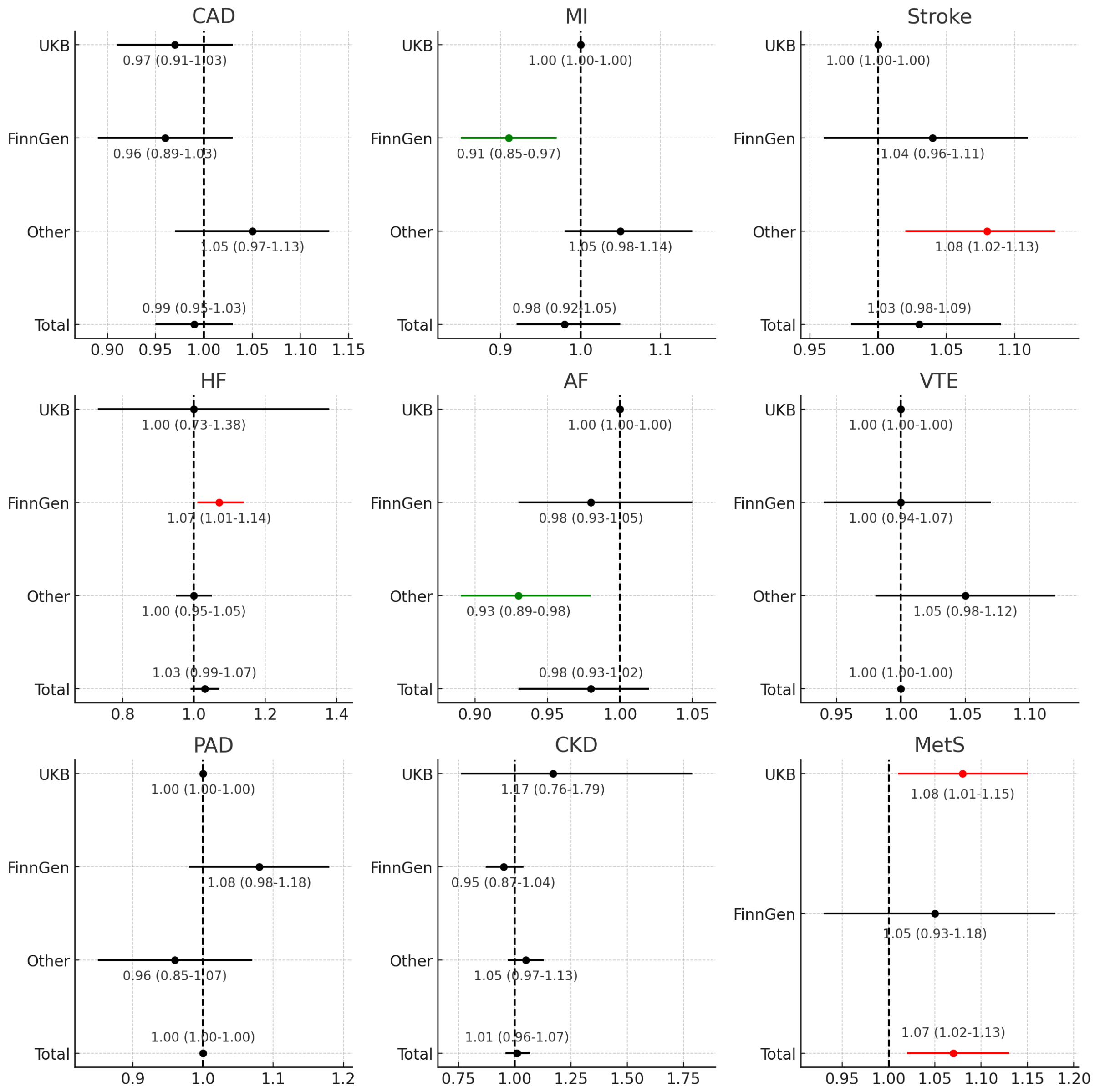

Figure S10. Pooled MR estimates of INSR (Insulin) on nine CKM syndrome‑related diseases.

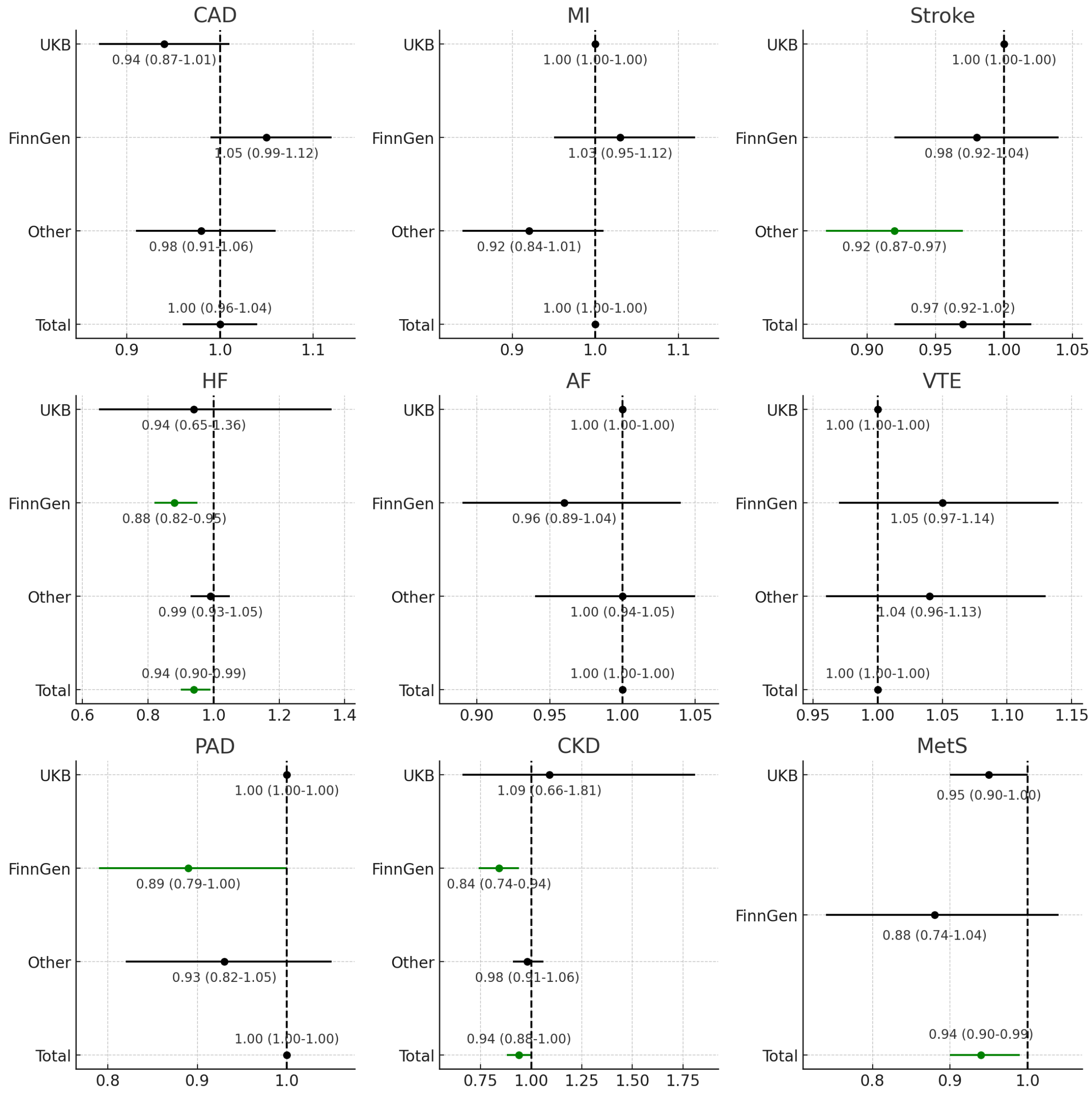

Figure S11. Pooled MR estimates of KCNJ11 (Sulfonylureas) on nine CKM syndrome－related diseases.

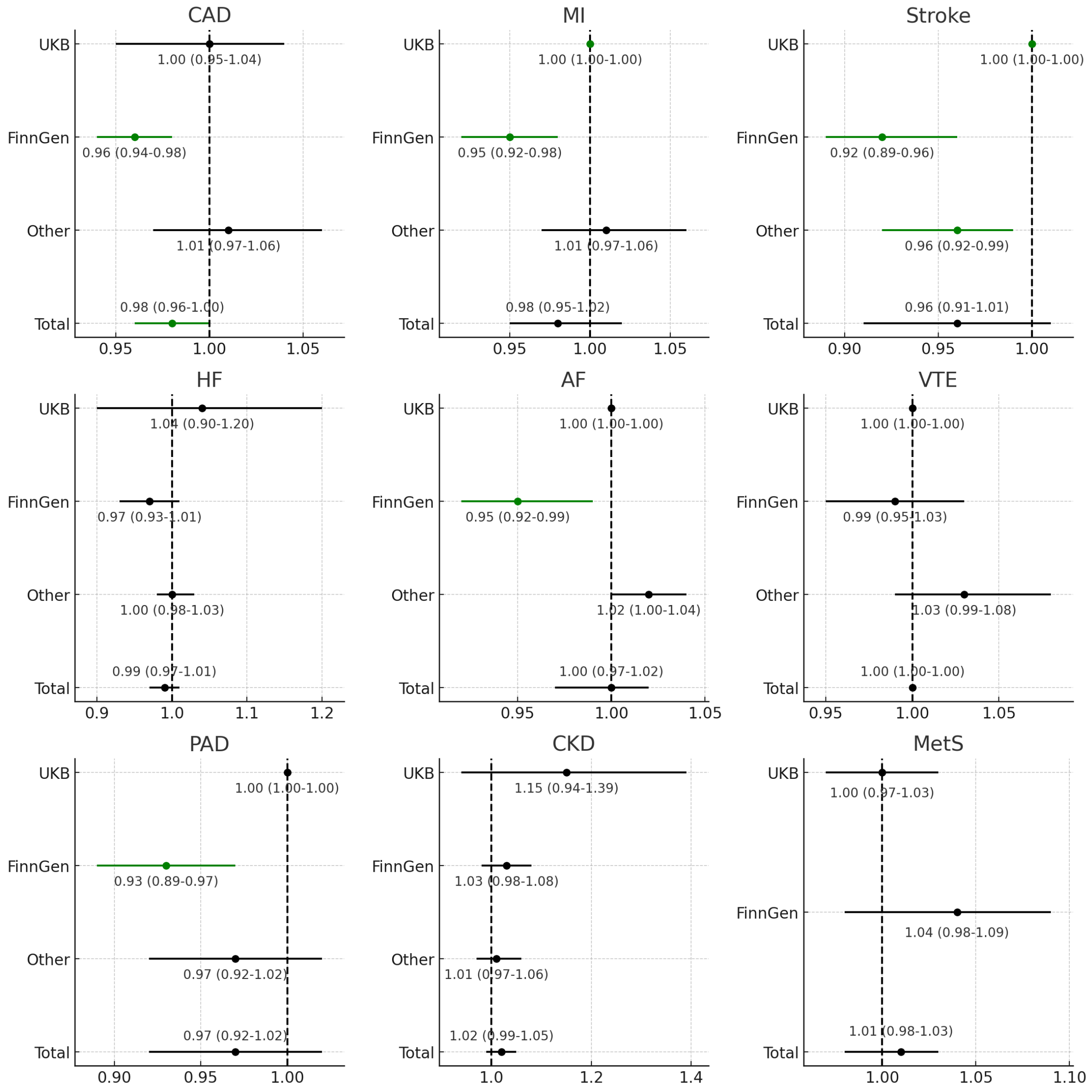

Figure S12. Pooled MR estimates of VEGFA (Sulfonylureas) on nine CKM syndrome-related diseases.

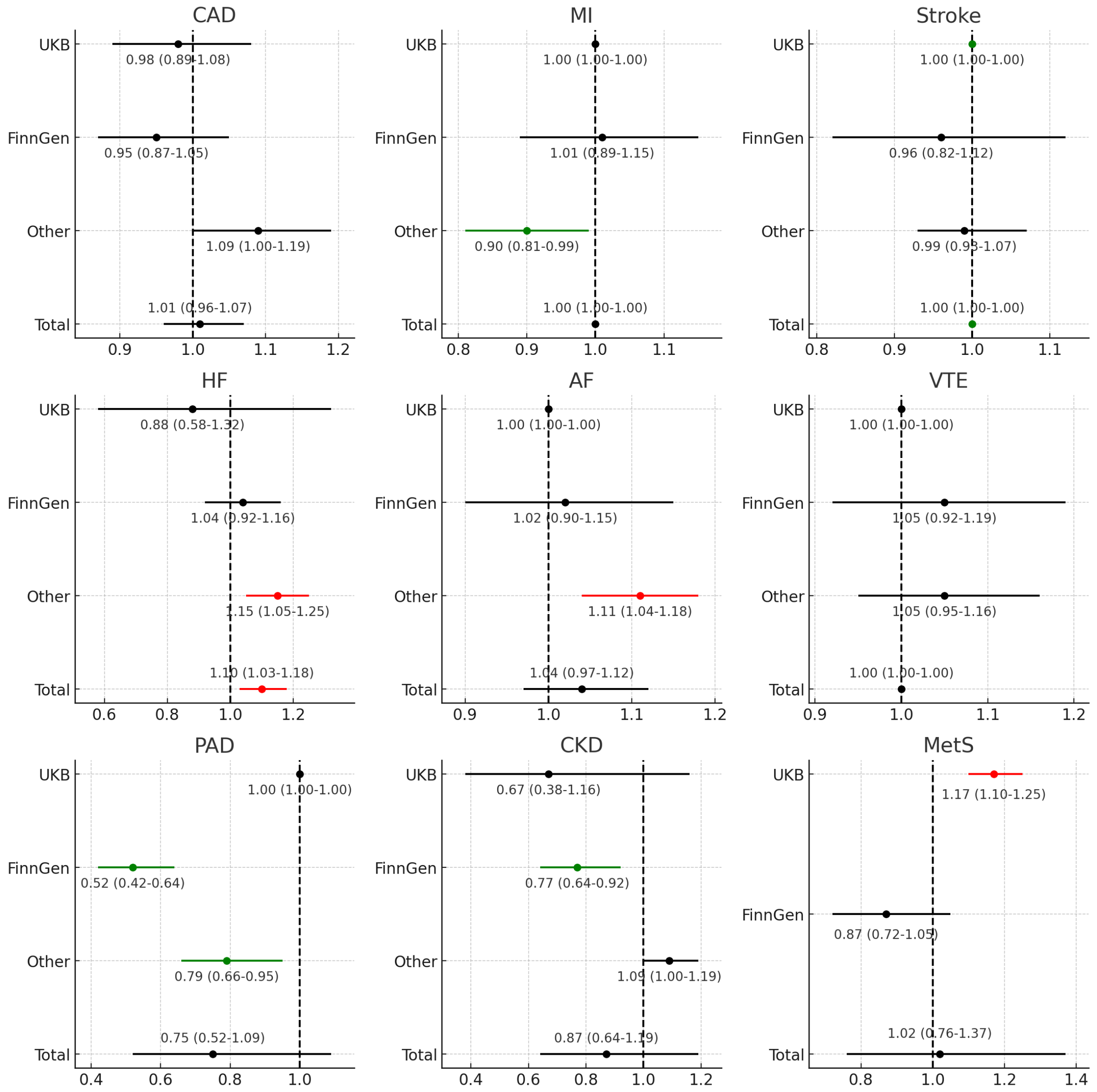

Figure S13. Pooled MR estimates of PPARD (TZDs) on nine CKM syndrome‑related diseases.

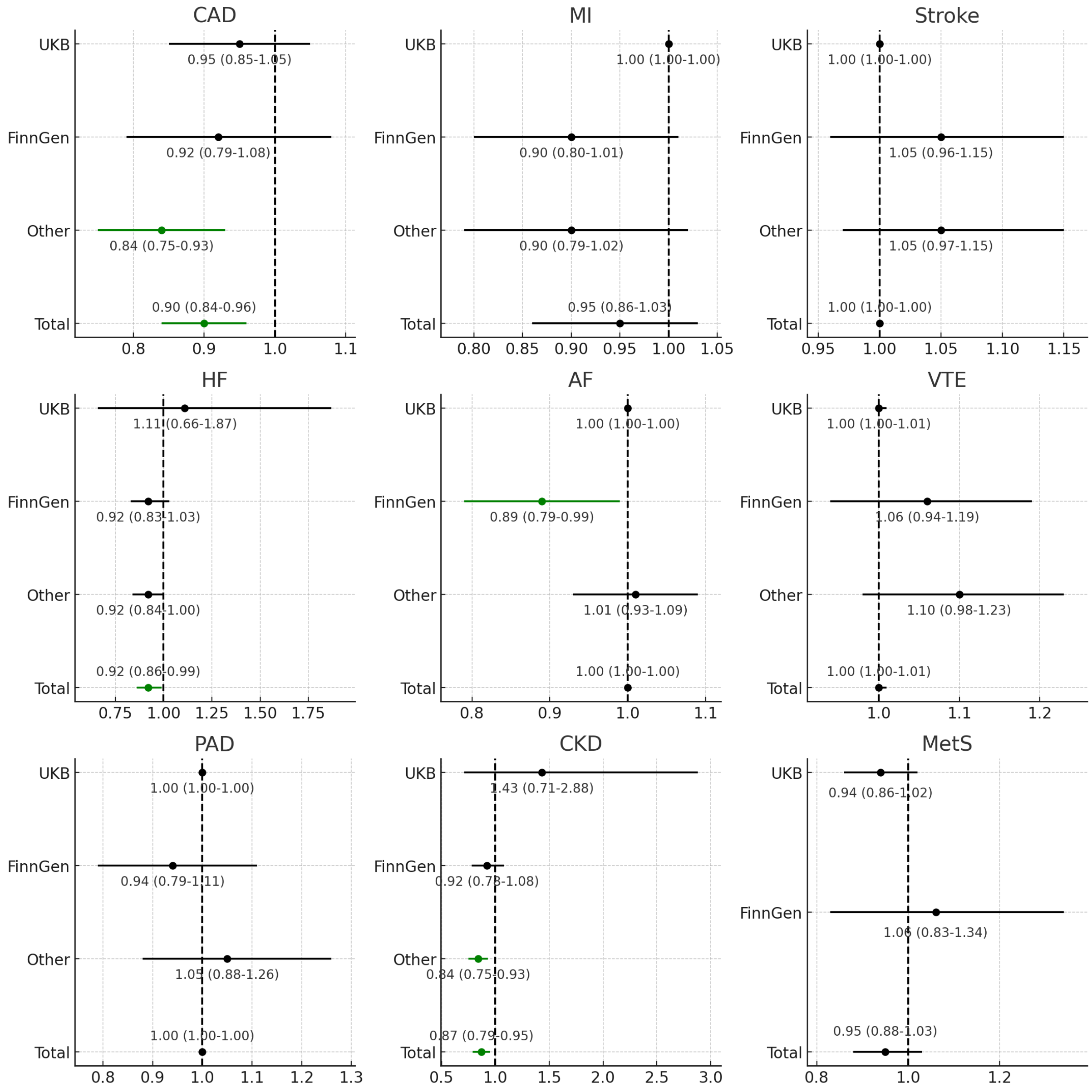

Figure S14. Pooled MR estimates of GLP1R (GLP-1RA) on nine CKM syndrome - related diseases.
